# Dronedarone produces early regression of myocardial remodelling in structural heart disease

**DOI:** 10.1371/journal.pone.0188442

**Published:** 2017-11-21

**Authors:** Begoña Quintana-Villamandos, Jose Juan Gomez de Diego, María Jesús Delgado-Martos, David Muñoz-Valverde, María Luisa Soto-Montenegro, Manuel Desco, Emilio Delgado-Baeza

**Affiliations:** 1 Departamento de Anestesiología, Hospital General Universitario Gregorio Marañón, Madrid, Spain; 2 Departamento de Farmacología, Facultad Medicina, Universidad Complutense de Madrid, Spain; 3 Departamento de Medicina y Cirugía Experimental, Instituto de Investigación Sanitaria Gregorio Marañón, Madrid, Spain; 4 Departamento de Cardiología, Hospital Clínico San Carlos, Madrid, Spain; Instituto de Investigación Sanitaria Gregorio Marañón, Madrid, Spain; 5 Departamento de Cirugía Experimental, Facultad Medicina, Universidad Autónoma de Madrid, Spain; 6 Departamento de Bioingeniería e Ingeniería Aeroespacial, Universidad Carlos III de Madrid, Spain; Medical College of Wisconsin, UNITED STATES

## Abstract

**Background and aims:**

Left ventricular hypertrophy (LVH) in hypertension is associated with a greater risk of sustained supraventricular/atrial arrhythmias. Dronedarone is an antiarrhythmic agent that was recently approved for the treatment of atrial fibrillation. However, its effect on early regression of LVH has not been reported. We tested the hypothesis that short-term administration of dronedarone induces early regression of LVH in spontaneously hypertensive rats (SHR_s_).

**Methods:**

Ten-month-old male SHR_s_ were randomly assigned to an intervention group (SHR-D), where animals received dronedarone treatment (100 mg/kg) for a period of 14 days, or to a control group (SHR) where rats were given vehicle. A third group with normotensive control rats (WKY) was also added. At the end of the treatment with dronedarone we studied the cardiac anatomy and function in all the rats using transthoracic echocardiogram, cardiac metabolism using the PET/CT study (2-deoxy-2[18F]fluoro-D-glucose) and cardiac structure by histological analysis of myocyte size and collagen content.

**Results:**

The hypertensive vehicle treated SHR rats developed the classic cardiac pattern of hypertensive cardiomyopathy as expected for the experimental model, with increases in left ventricular wall thickness, a metabolic shift towards an increase in glucose use and increases in myocyte and collagen content. However, the SHR-D rats showed statistically significant lower values in comparison to SHR group for septal wall thickness, posterior wall thickness, ventricular mass, glucose myocardial uptake, size of left ventricular cardiomyocytes and collagen content. All these values obtained in SHR-D rats were similar to the values measured in the normotensive WKY control group.

**Conclusion:**

The results suggest by three alternative and complementary ways (analysis of anatomy and cardiac function, metabolism and histological structure) that dronedarone has the potential to reverse the LVH induced by arterial hypertension in the SHR model of compensated ventricular hypertrophy.

## Introduction

Arterial hypertension is the main cause for the most frequent sustained arrhythmia in clinical practice, atrial fibrillation. Antihypertensive treatment has the potential to restore blood pressure levels, reduce the hypertrophy induced by chronic hypertension (left ventricular hypertrophy, LVH) and prevent the development of atrial fibrillation [1–3]. Regression of LVH has been reported after long-term treatment with antihypertensive drugs [4,5]. In a preclinical study, we previously analyzed early regression of LVH following short-term use of antihypertensive agents and found that esmolol produces regression of LVH after 2 days of intravenous treatment [6].

Dronedarone is an antiarrhythmic agent that was recently approved for the treatment of atrial fibrillation (AF) [7]. It reduces the incidence of recurrences of AF, cardiovascular-related hospitalizations, and death in patients with paroxysmal or persistent AF; however, it is avoided in high-risk patients with permanent AF or patients with unstable chronic heart failure [8,9]. The complete mechanism of action of dronedarone is unknown, although we do know that the drug exerts its antiarrhythmic effects through multichannel blockade of the sodium, potassium, and calcium channels and exhibits antiadrenegic properties [10]. Dronedarone is the first antiarrhythmic drug that has been proven to reduce cardiovascular morbidity and mortality in clinically stable patients with other risk factors for recurrent AF [11]. However, its effect on rapid regression of LVH in compensated structural heart disease has not yet been studied.

The objective of the present work is to study whether dronedarone, has the potential to modify the cardiac alterations caused by arterial hypertension (LVH) in 10-month-old spontaneously hypertensive rats (SHRs) (model of compensated LVH).

## Materials and methods

All procedures fulfilled the stipulations of the Guidelines for the Care and Use of Laboratory Animals (Directive 2010/63/EU and Spanish Law RD 53/2013) and were approved by the Ethics Committee of Hospital General Universitario Gregorio Marañon, Madrid, Spain (PROEX 223/14).

### Animals and experimental protocols

The study animals—10-month-old male SHRs (n = 18) and normotensive control Wistar-Kyoto (WKY) rats (n = 9)—were bred at the animal house of Universidad Autonoma de Madrid. All the rats were supplied with standard rat chow and drinking water ad libitum and were maintained on a 12 h/12 h light/dark cycle. The animals were housed at a constant temperature of 24°C and relative humidity of 40%. SHRs were randomly divided into two groups (9 rats each): dronedarone-treated rats (SHR-D) and vehicle-treated rats (SHR), which was the control group for the SHR-D. The SHR-D received oral dronedarone 100 mg/kg once daily for 14 days. Control SHR and WKY received saline solution (vehicle). Once treatment was complete, transthoracic echocardiography was used to study morphology and cardiac function, and PET/CT scans were carried out to study cardiac glucose metabolism. Rats were killed by decapitation after sedation with an intraperitoneal injection of diazepam 10 mg/kg and ketamine 80 mg/kg and a histological analysis of heart tissue was performed.

### Blood pressure and heart rate measurements

Systolic arterial pressure (SAP) and heart rate (HR) were measured (conscious animals prewarmed to 35°C in thermostatic cages) using the tail-cuff method with a photoelectric sensor (Niprem 546, Cibertec, Madrid, Spain). Several determinations were made, and the findings were considered valid if 10 consecutive measurements were within 10 mmHg of each other.

### Echocardiographic studies

Transthoracic echocardiography was performed using the VIVID q system (GE Healthcare, Germany) equipped with a 13-MHz probe (12S-RS, GE). The images were acquired with the animals in left lateral decubitus. Transthoracic echocardiography was performed under anaesthesia (intraperitoneal diazepam 10 mg/kg and ketamine 80 mg/kg) after 14 days of treatment. Values were determined by averaging the measurements of 3 consecutive cardiac cycles in accordance with American Society of Echocardiography guidelines [12]. M-mode imaging of the parasternal short axis (papillary level) enabled the measurement of LV end systolic and end diastolic internal dimensions (LVIDs and LVIDd, respectively), posterior wall diastolic thickness (PWd), and interventricular septal end diastolic thickness (IVSd). The above measurements were used for calculations of left ventricular mass (LVM) as previously described [6] based on the following equation:

$$LVM=0.8\times [1.04\times {\left(IVSd+LVIDd+PWd\right)}^{3}-{\left(LVIDd\right)}^{3}]+0.6g$$

LVM was adjusted for body weight by calculating the left ventricular mass index (LVMI). Transthoracic echocardiography also enabled the evaluation of relative wall thickness (RWT) [6]. LV ejection fraction and fractional shortening were calculated as measures of LV systolic function, as previously described [6]. In addition, the pulsed-wave Doppler early-to-late transmitral peak diastolic flow velocity ratio (E/A ratio) was measured to assess diastolic function (E, mitral peak early-filling velocity; and A, mitral peak flow velocity at atrial contraction). The transmitral flow velocity profile was determined by positioning a sample volume at the tip of the mitral valve on the para-apical long-axis view. The E-wave deceleration time was measured as the time interval between peak E-wave velocity and the point where the descending E-wave (or its extrapolation) intercepted the zero line [6].

### Cardiac PET/CT

Animals were scanned on a small-animal PET scanner (ARGUS PET-CT, SEDECAL, Madrid, Spain) under anaesthesia (sevoflurane 5% for induction and 2% for maintenance in 100% oxygen). Approximately 1 mCi of 2-deoxy-2[18F]fluoro-D-glucose (^18^F-FDG) (Molypharma, Madrid, Spain) was injected into the tail vein and, after an uptake period of 45 minutes, animals were scanned for 45 minutes. Blood glucose levels were measured before and after PET acquisition using blood strips (Accu-Chek Performa, Roche). Images were reconstructed using the 2D-OSEM (ordered subset expectation maximization) algorithm, which yields a spatial resolution for this scanner of 1.45 mm full width at half maximum (FWHM), with a voxel size of 0.3875 × 0.3875 × 0.7750 mm^3^. The energy window was 400–700 keV. Decay and deadtime corrections were applied.

PET data were analyzed using regions of interest (ROI) in the multimodality workstation provided in the PET scanner. The ROIs drawn on the coronal, axial, and sagittal sections were heart and muscle (dorsal muscle). The PET data assessment included analysis of the mean standardized uptake value (SUV), which takes into account tissue radioactivity concentration, injected activity, and body weight. The SUV for each ROI (heart, muscle) and the heart-to-muscle ratio were used for analysis, as previously described [13].

### Morphometric analysis of LV and atria

After the echocardiogram, the heart was excised, weighed, and fixed in 4% paraformaldehyde. A equatorial cross-section of the LV was embedded in paraffin. Sections were cut (at a thickness of 5-μm) and stained with hematoxylin-eosin. The cross-sectional area (CSA) of cardiomyocytes was measured in the subepicardium (~ 25 cardiomyocytes measured in each picture and 3 pictures per region, giving a total of ~ 150 cardiomyocytes measured in each LV), as described elsewhere [6]. The extent of fibrosis was determined as described [14]. For collagen staining, 5-μm sections (5 sections/rat) of paraffin blocks were stained with picrosirius red. Images were captures using a high-resolution camera (Sony CCD IRIS) attached to a microscope (Leica DMLB, x4 objective). The image analysis was performed using the method of Gundersen et al. [15]. The images were projected on a computer screen (5 images of each section) and, for each image, the collagen volume fraction (CVF) was determined as the ratio of points on the birefringent collagen fibres with respect to points on the remaining myocardium. A sagital cross-section of the atria was embedded in paraffin. Sections were cut (at a thickness of 5-μm) and stained with picrosirius red. The extent of fibrosis was determined as described above.

### Statistical analysis

The primary endpoint was LVM in SHRs after 14 days administration of dronedarone, which was compared between groups. The variable was expressed as mean ± SEM. We used the Kolmogorov-Smirnov test to analyze the distribution of quantitative variables. The parameters were compared using single-factor (rat) analysis of variance (physiological, echocardiographic, PET/CT, and histological parameters). A post hoc Bonferroni correction was applied. Statistical significance was set at P ≤ 0.05. The analysis was performed using IBM SPSS Statistics for Windows, version 20.0 (IBM Corp, Armonk, New York, USA).

## Results

### Physiological parameters

Rat weight was significantly greater in WKY than in SHR (467.40 ± 4.37 vs. 398.93 ± 8.43 g, P < 0.01), although no statistically significant differences were detected between SHR and SHR-D (398.93 ± 8.43 vs. 363.46 ± 4.57 g). Dronedarone lowered SAP in SHR-D with respect to control SHR (142 ± 10 vs. 181 ± 11 mmHg, P < 0.01), and SAP was comparable to that of the WKY (142 ± 10 vs. 130 ± 10 mmHg). Dronedarone lowered HR in SHR-D with respect to the WKY and SHR controls (279 ± 19 vs. 414 ± 10 and 410 ± 18 beats/min, P < 0.001).

### Echocardiographic parameters

**Fig 1:**
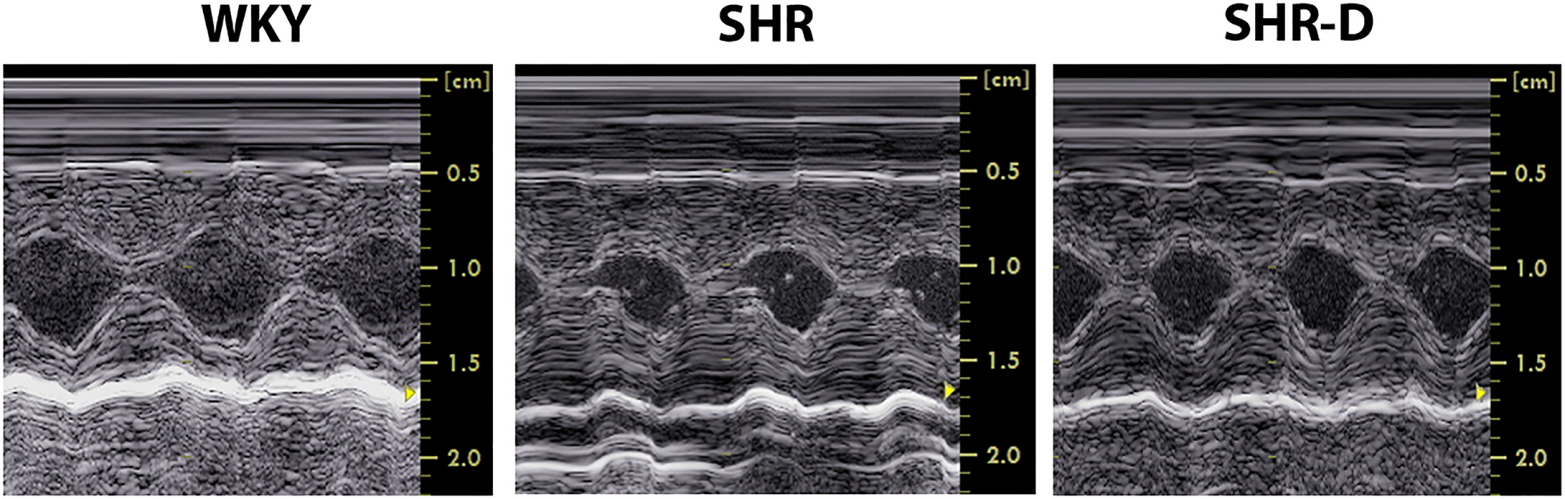
Left ventricular geometry obtained by echocardiography. Examples of M-mode echocardiograms from a Wistar-Kyoto rat treated with vehicle (WKY), a spontaneously hypertensive rat treated with vehicle (SHR) and a spontaneously hypertensive rat treated with dronedarone (SHR-D).

Left ventricular geometry indices obtained using M-mode echocardiography are shown in Table 1 and Fig 1. SHR presented concentric LVH associated with an increase of LVMI (45.8%, P < 0.05) and RWT (34.7%, P < 0.05) when compared with WKY. Compared to untreated SHR, dronedarone administration to SHR-D resulted in a decrease of LVMI (35.8%, P < 0.05) and RWT (30.6%, P < 0.01) and no differences were detected with respect to WKY. IVSd and PWd of SHR in comparison with WKY increased by 37.3% (P < 0.001) and 44.5% (P < 0.001). Two weeks of dronedarone administration to SHR-D decreased IVSd by 28.1% (P < 0.001) and PWd by 21.3% (P < 0.01) and no differences were detected with respect to WKY.

**Table 1:** Echocardiographic parameters with M-mode and transmitral inflow measurements.

	WKY (*n* = 9)	SHR (*n* = 9)	SHR-D (*n* = 9)	P-values ANOVA
LVIDd (mm)	5.06 ± 0.17	5.50 ± 0.43	5.89 ± 0.33	0.232
LVIDs (mm)	2.44 ± 0.13	2.07 ± 0.34	2.43 ± 0.35	0.602
IVSd (mm)	2.46 ± 0.09	3.38 ± 0.14***	2.43 ± 0.08 ^†††^	<0.001
PWd (mm)	2.20 ± 0.11	3.18 ± 0.11***	2.50 ± 0.14 ^**††**^	<0.001
LVMI (mg/g)	2.68 ± 0.02	3.91 ± 0.57*	2.51 ± 0.13^†^	0.015
RWT	0.92 ± 0.04	1.24 ± 0.09*	0.86 ± 0.06^††^	0.002
EF (%)	77.04 ± 1.20	85.17 ± 2.83	82.40 ± 3.08	0.088
FS (%)	52.22 ± 1.26	63.53 ± 4.00	58.32 ± 4.20	0.086
E-wave (cm/s)	0.77 ± 0.06	0.69 ± 0.07	0.54 ± 0.05	0.063
A-wave (cm/s)	0.46 ± 0.04	0.48 ± 0.04	0.37 ± 0.04	0.194
*E/A* ratio	1.72 ± 0.10	1.52 ± 0.25	1.57 ± 0.20	0.760
Edec time (ms)	40 ± 8	31 ± 1	42 ± 4	0.352

LVIDd, left ventricular end diastolic diameter; LVIDs, left ventricular end systolic diameter; IVSd, interventricular septal end diastolic thickness; PWd, left ventricular posterior wall diastolic thickness; LVMI, left ventricular mass index; RWT, relative wall thickness; EF, left ventricular ejection fraction; FS, left ventricular fractional shortening; E-wave, mitral peak early-filling velocity; A-wave, mitral peak flow velocity at atrial contraction; E/A, early-to-atrial filling velocity ratio; Edec time, E-wave deceleration time; WKY, Wistar-Kyoto rats treated with vehicle; SHR, spontaneously hypertensive rats treated with vehicle; SHR-D, spontaneously hypertensive rats treated with dronedarone; Statistically significant differences between WKY, SHR and SHR-D are shown

*P<0.05 vs. WKY

***P<0.001 vs. WKY

^†^P<0.05 vs. SHR

^††^P<0.01 vs. SHR

^†††^P<0.001 vs. SHR.

Values are given as mean ± SEM (n = 9 rats per group).

Two indicators of systolic function, FS and EF, were similar in all three groups (WKY, SHR, SHR-D) (Table 1). There were no significant differences in the E/A ratio, E-wave, A-wave or deceleration time (data for diastolic function) in WKY, SHR, and SHR-D (Table 1).

### PET measurement of ^18^F-FDG (myocardial glucose metabolism)

**Fig 2:**
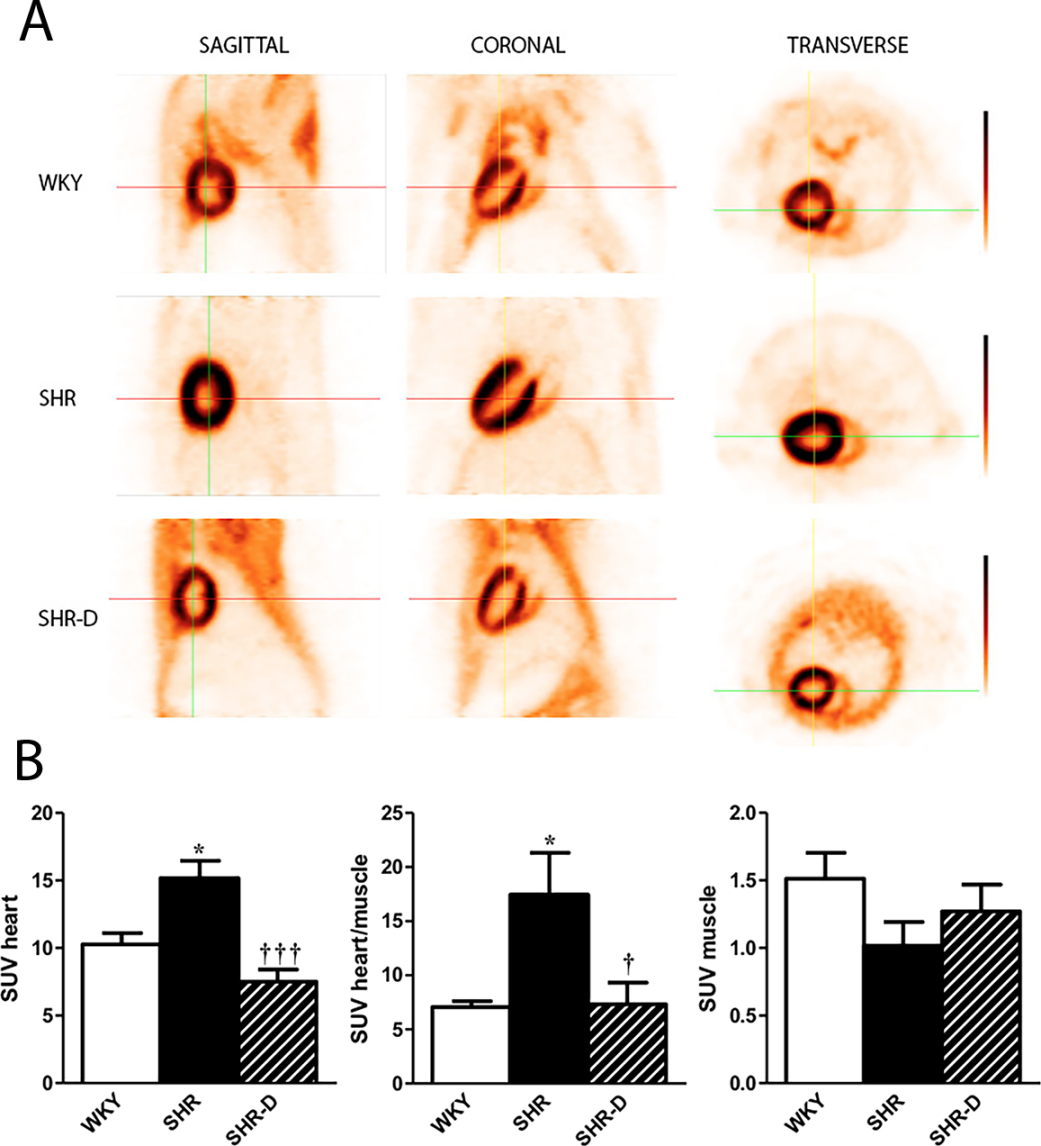
Cardiac glucose metabolism obtained by positron emission tomography. PET/CT images of myocardial 18 F-FDG uptake from a Wistar-Kyoto rat treated with vehicle (WKY), a spontaneously hypertensive rat treated with vehicle (SHR) and a spontaneously hypertensive rat treated with dronedarone (SHR-D) (A). Dronedarone produced a marked decrease in the glucose metabolism of the hypertrophied ventricle. There were no significant differences in standardized uptake value (SUV_heart_) in either SHR-D or WKY. Statistically significant differences between WKY, SHR, and SHR-D are shown (*P<0.05 vs. WKY; ^†^P<0.05 vs. SHR; ^†††^P<0.001 vs. SHR). Values are given as mean ± SEM (n = 6 rats per group) (B).

The SUV_mean_ for heart, muscle, and heart/muscle ratio is shown in Fig 2A and 2B. SUV _heart_ of SHR in comparison with WKY increased by 47.6% (P < 0.05). The quantitative analysis (SUV_heart_) shows that dronedarone produced a marked decrease in the glucose metabolism (50.4%, P < 0.001) of the hypertrophied ventricle (SHR-D) with respect to SHR. There were no significant differences in SUV_heart_ in either SHR-D or WKY.

### Histological parameters

The heart weight (HW) in SHR group was increased by 20.1% (P < 0.05) when compared to WKY. Dronedarone administration resulted in a decrease of HW in SHR-D by 23.5% (P < 0.01). No differences in HW were found between WKY and SHR receiving dronedarone (Table 2).

**Table 2:** Histological variables of the left ventricle and atria.

	WKY (n = 6)	SHR (n = 6)	SHR-D (n = 6)	P-values ANOVA
HW (g)	1.59 ± 0.07	1.91 ± 0.09*	1.46 ± 0.07^††^	0.003
CSA (μm^2^) Subepicardium	100 ± 8	275 ± 50***	70 ± 7^†††^	<0.001
CVF (%) Subepicardium	1.05 ± 0.21	4.31 ± 0.63***	1.34 ± 0.24^†††^	<0.001
CVF (%) atria	3.59 ± 0.28	7.95 ± 0.32***	5.3 ± 0.24**^,^^†††^	<0.001

HW, heart weight; CSA, cross-sectional area of cardiomyocytes; CVF, collagen volume fraction; WKY, Wistar-Kyoto rat treated with vehicle; SHR, spontaneously hypertensive rat treated with vehicle; SHR-D, spontaneously hypertensive rats treated with dronedarone. Statistically significant differences between WKY, SHR, and SHR-D are shown

*P<0.05 vs. WKY

**P<0.01 vs. WKY

***P<0.001 vs. WKY

^††^P<0.01 vs. SHR

^†††^P< 0.001 vs. SHR.

Values are given as mean ± SEM (n = 6 rats per group).

**Fig 3:**
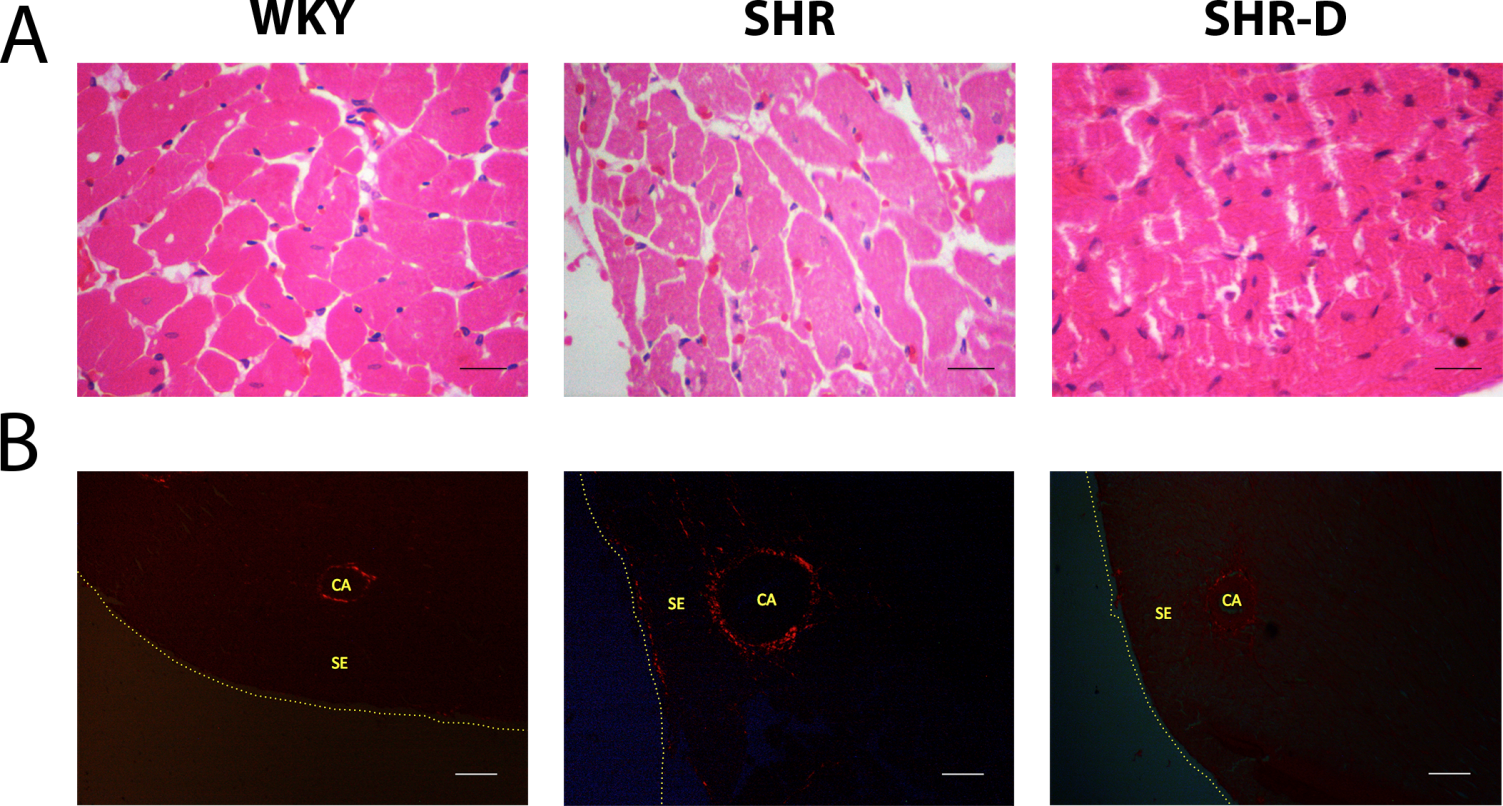
Examples of histological sections of the left ventricle. Examples of histological sections of the subepicardial region of the left ventricle from a Wistar-Kyoto rat treated with vehicle (WKY), a spontaneously hypertensive rat treated with vehicle (SHR) and a spontaneously hypertensive rat treated with dronedarone (SHR-D), H&E (x40 objective; scale bar = 20 μm)(A) and Pricosirius red (CA, coronary artery; SE, subepicardium) (x4 objective; scale bar = 150 μm)(B).

The CSA of cardiomyocytes and CVF of left ventricle are shown in Table 2 and Fig 3A and 3B. CSA and CVF in SHR in comparison with WKY increased by 175% (P < 0.001) and 310.4% (P < 0.001). Compared to untreated SHR, dronedarone administration to SHR-D resulted in a decrease of CSA and CVF (74.5%, P < 0.001 and 68.9%, P < 0.001 respectively). No significant differences in CSA and CVF were observed in SHR-D compared with WKY.

**Fig 4:**
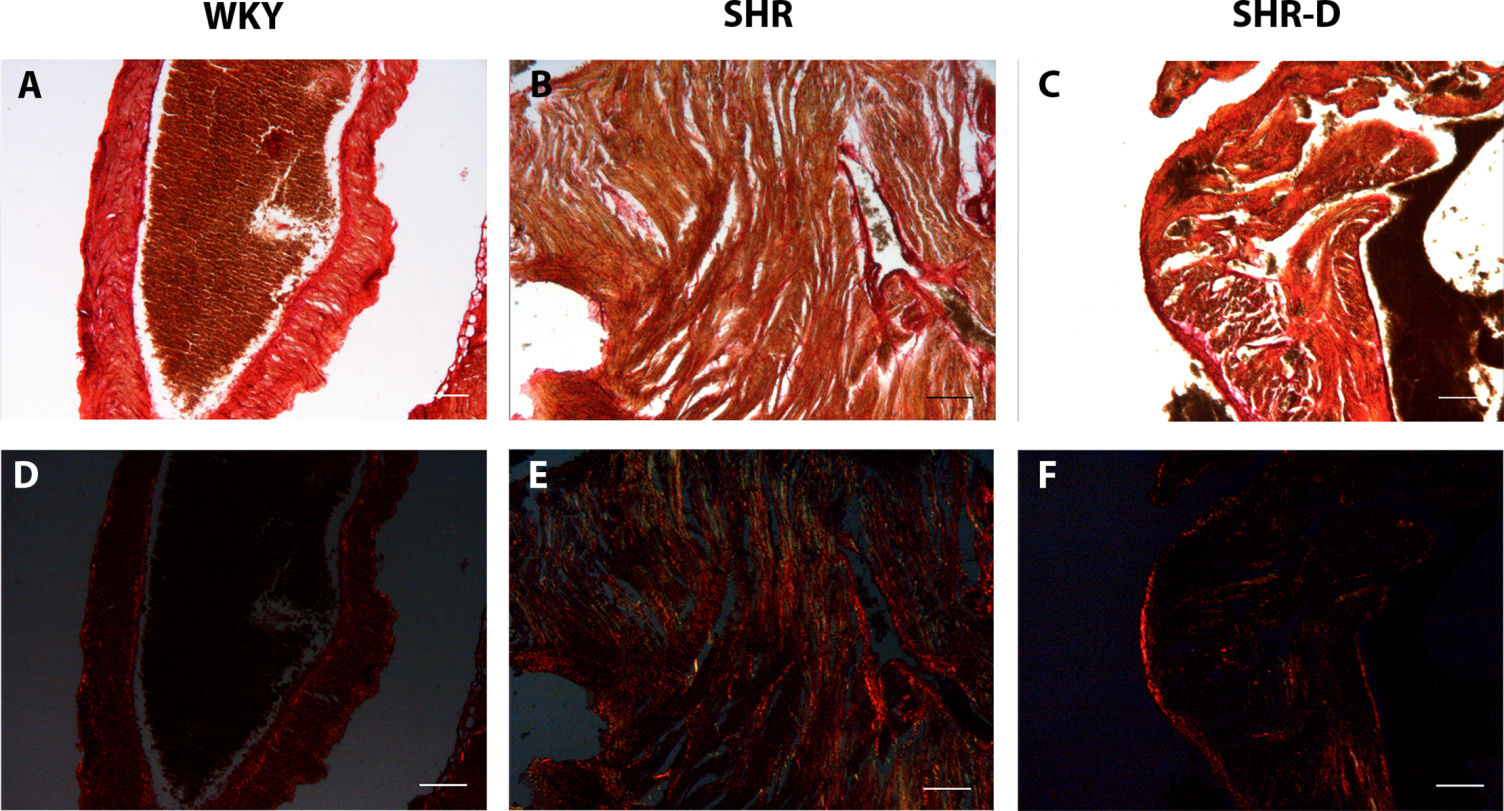
Examples of histological sections of the atria. Examples of histological sections of the atria from a Wistar-Kyoto rat treated with vehicle (WKY), a spontaneously hypertensive rat treated with vehicle (SHR) and a spontaneously hypertensive rat treated with dronedarone (SHR-D), Pricosirius red (x4 objective; scale bar = 150 μm).

The CVF of atria is shown in Table 2 and Fig 4. CVF was increased in SHR (121.4%, P < 0.001) when compared with WKY. CVF in SHR-D was smaller (33.3%, P < 0.001) than that in SHR and the difference was also significant in comparison with WKY rats (P < 0.01).

## Discussion

Our results suggest by three alternative and complementary ways (analysis of anatomy and cardiac function, myocardial glucose metabolism and histological structure) that short-term treatment (14 days) with dronedarone (100 mg/kg orally and once daily) has the potential to reverse the hypertrophy induced by chronic hypertension in the SHR model of compensated ventricular hypertrophy.

### Effect of dronedarone on SAP, HR, and cardiac function

Dronedarone is a class III antiarrhythmic drug, although it shares effects with all 4 Vaughan-Williams classes of antiarrhythmics (sodium channel blockers, beta and alpha-blockers, potassium channel blockers, and nondihydropyridine calcium channel blockers) [10]. The efficacy of dronedarone for the maintenance of sinus rhythm and control of ventricular rate has been demonstrated in randomized clinical trials [11]. In the present study, dronedarone led to a reduction in HR and SAP in SHRs. In preclinical studies, dronedarone decreased HR (by direct inhibition of the hyperpolarization-activated “funny” current [I_f_]) [16] and mean arterial blood pressure (by inhibiting α-adrenoceptor-mediated vasoconstriction) [17].

Dronedarone has a negative inotropic effect on the myocardium [18]. This effect likely underlies the increased mortality associated with dronedarone in patients with severe heart failure and systolic dysfunction [19]. However, our results are consistent with those of other authors [16,20], in that administration of dronedarone did not produce alterations in ventricular function parameters measured noninvasively with the echocardiogram under resting conditions, perhaps because we used a compensated hypertrophy model (SHRs aged 10 months).

### Effect of dronedarone on LVH

Regression of LVH has been reported after long-term antihypertensive treatments (lisinopril over three months, candesartan over one year and captopril over five weeks in SHR) [4,5, 21–23]. However, in a preclinical study, we previously showed that esmolol (β-adrenergic blocker) produces regression of LVH after two days of intravenous treatment [6]. In a model of thyroxine-induced cardiac hypertrophy in rats, Pantos et al.[24] did not observe a decrease in LVM after treatment with dronedarone, perhaps because the dose was a third of what we used in the present study (30 mg/kg for two weeks once daily).

In our study, oral dronedarone reduced LVM after short-term treatment (14 days), and this finding coincided with changes in the cardiomyocytes and collagen of the LV. The mechanism responsible for this effect is unknown. Nitric oxide (NO) is a key regulator of cardiovascular remodelling. Left ventricular mass is strongly modulated by the nitric oxide system. Low levels of NO resulting from reduced nitric oxide synthase induce LVH [25,26], and the regression of LVH has been related to an increase in nitric oxide synthase and increased bioavailability of NO [27,28]. Dronedarone produces a coronary dilation by stimulation of nitric oxide synthase pathway (this effect appears to be due to a release of NO) [29]. Therefore, stimulation of the NO pathway by dronedarone could explain in part its effect on the regression of LVH.

### Effect of dronedarone on myocardial glucose metabolism

The primary energy source in the heart are fatty acids [30], however, glucose is more important in cardiovascular pathology (LVH) [31,32]. Our group observed an increase in myocardial glucose metabolism in SHR [13] and other studies have shown similar results in other animal models of cardiac hypertrophy (salt-sensitive Dahl rats, Wistar rats with ascending aortic constriction) [33–35].

Antihypertensive treatment reduces the hypertrophy induced by chronic hypertension and prevents the development of cardiovascular events [36,37]. The reduction of LVM and an improvement in cardiac metabolism are two complementary signs of the regression og LVH [1,38]. In the present study, in addition to reduced LVM, it is noteworthy that myocardial glucose decreased considerably after 14 days of treatment with dronedarone. This result was similar to those of a preclinical study in which our group showed changes in myocardial glucose metabolism in early regression of LVH after only 48 h of treatment with a beta-blocker (esmolol) [6].

### Dronedarone and clinical implications

The present study provides preclinical data on the beneficial effects of dronedarone on regression of LVH, which is associated with a high risk of sustained supraventricular/trial arrhythmias in hypertensive patients [39]. Antihypertensive treatment has the potential to restore blood pressure levels, reduce the hypertrophy induced by chronic hypertension and prevent the development of atrial fibrillation. Antiarrhythmic drugs have the potential to reduce the incidence of sudden death in patients with heart disease [24]. Dronedarone is contraindicated in patients with New York Heart Association (NYHA) class IV heart failure or in NYHA class II-III heart failure with a recent decompensation because of a possible increase in mortality [11]. However, we studied the effect of short-term administration of dronedarone in an experimental rat model of compensated ventricular hypertrophy (10-month-old SHR) [40,41].

If the results of this study are confirmed in humans, this effect should be very interesting in clinical practice in patients with hypertrophy induced by chronic hypertension. LVH regression might reduce the incidence of cardiac arrhythmias [3]. If dronedarone causes a decrease in left ventricular mass, perhaps this decrease could potentiate the antiarrhythmic effect of dronedarone in atrial fibrillation treatment. The present study shows the decrease of collagen deposition in the atria after treatment with dronedarone. These data could support the hypothesis that dronedarone produces regression cardiac remodelling and this regression could be related to the antiarrhythmic effect of the drug in atrial fibrillation. However, we need further studies to demonstrate this claim.

### Limitations

Our study is subject to a series of limitations. First, cardiac remodelling involves various sets of genes during regression and during induction of hypertrophy [37]. Consequently, the beneficial medium-term effect of the regression of LVH does not mean that the original genotype will be re-expressed and the myocardial structure will return to normal [2]. Therefore, the regression of LVH by dronedarone does not guarantee that the heart will be normal in all aspects. Second, the mechanisms underlying this effect (regression of LVH) following short-term use have not been analyzed in the present study. Although the effect could be attributed in part to stimulation of the NO pathway by dronedarone [29]. Further investigations are needed to explain the regression of LVH with this drug.

## Conclusions

Our results suggest by three alternative and complementary ways (analysis of anatomy and cardiac function, metabolism and histological structure) that dronedarone has the potential to reverse the hypertrophy induced by chronic hypertension in the SHR model of compensated ventricular hypertrophy.
